# Supplementary material for: MicroRNA-27a controls the intracellular survival of Mycobacterium tuberculosis by regulating calcium-associated autophagy
Source: Nat Commun. 2018 Oct 16;9:4295. doi: 10.1038/s41467-018-06836-4 (PMC6191460; doi:10.1038/s41467-018-06836-4)
Supplement: Supplementary file 1 — Supplementary Information [file 41467_2018_6836_MOESM1_ESM.pdf]

Supplementary Materials

MicroRNA-27a controls the intracellular survival of *Mycobacterium tuberculosis* by regulating calcium-associated autophagy

Liu *et al*.,

Supplementary Figures and Figure legends 1-16

Supplementary Table1 Realtime PCR Primers

Supplementary Table2 SiRNA sequence

Supplementary Figures and Figure Legends

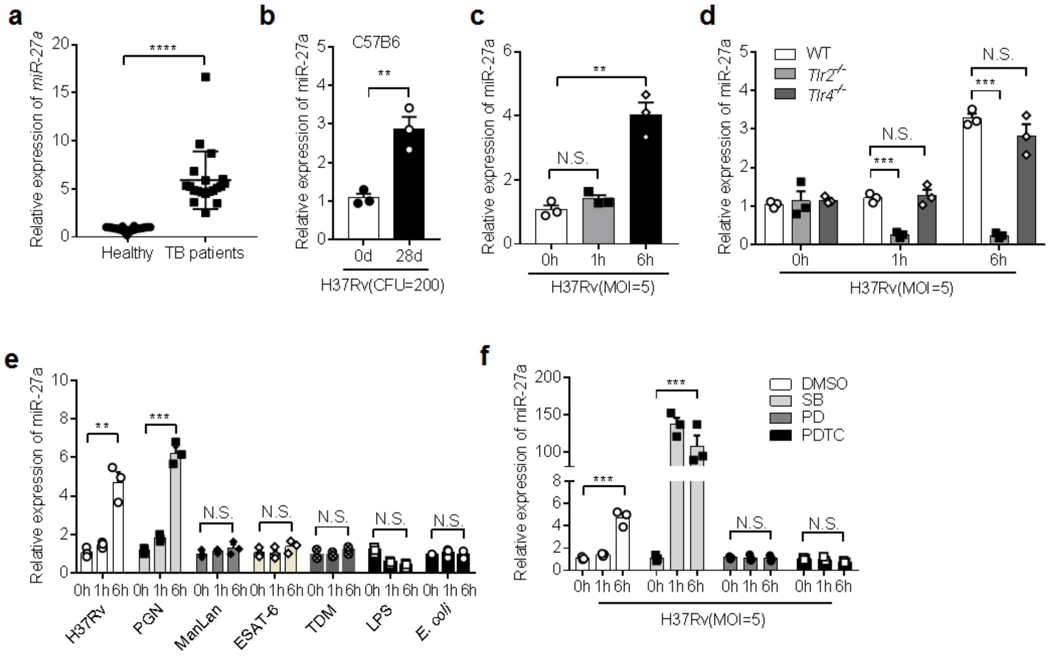

Supplementary Figure 1 Induction of miR-27a by *Mtb* infection (**a-f**) RT-PCR of *miR-27a* in: (**a**) PBMCs from healthy donors (n=21) and active TB patients (n=21); (**b**) Lung of mice (n=5) infected with H37Rv as in **b**; (**c**) Macrophages infected with H37Rv as in **c**. (**d**) WT, *Tlr2*$^{-/-}$, or *Tlr4*$^{-/-}$ macrophages infected with H37Rv; (**e**) Real-time PCR detection of miR-27a in murine primary peritoneal macrophages stimulated with H37Rv, PGN, ESAT6, ManLAM, TDM, LPS and *E. coli* for indicated times. (**f**) Macrophages pretreated with DMSO、SB203580 (10 μM)、PD98059 (10 μM) and PDTC (10 μM) for 1 hour, and then infected with H37Rv.* $p < 0.05$, ** $p < 0.01$, *** $p < 0.001$ and N.S., not significant ($p > 0.05$) by the unpaired *t*-test (**a-f**). Data are from representative of three independent experiments.

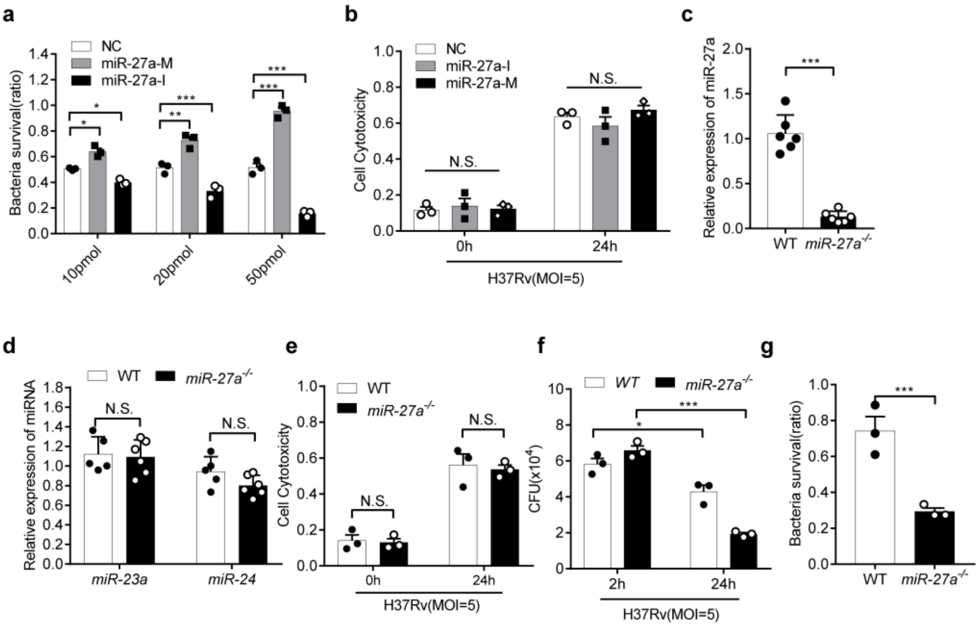

Supplementary Figure 2 Regulation of intracellular survival of *Mtb* by miR-27a

(**a**)CFU assay in primary peritoneal macrophages transfected with NC, miR-27a mimics or miR-27a inhibitors at indicated dose, and then infected with *Mtb* H37Rv (MOI=5) for 2 hours or 24 hours. (**b**) LDH assay in primary peritoneal macrophages transfected with NC, miR-27a mimics or miR-27a inhibitors and then infected with *Mtb* H37Rv (MOI=5) for 24 hours. (**c**) Real-time PCR detection of miR-27a in lung of WT or miR-27a KO mice. (**d**) Real-time PCR detection of miR-23a and miR-24 in macrophages of WT or miR-27a KO mice.(e)LDH assay in primary peritoneal macrophages from WT or *miR-27a*$^{-/-}$ mice infected with *Mtb* H37Rv (MOI=5) for 24 hours. (**f**) CFU assay in primary peritoneal macrophages from WT or *miR-27a*$^{-/-}$ mice infected with *Mtb* (MOI=5) for 2 hours or 24 hours. (**g**) Bacterial killing of peritoneal macrophages from WT or *miR-27a*$^{-/-}$ mice infected with *Mtb* (MOI=5) at 24 hours compared to 2 hours. * $p < 0.05$, ** $p < 0.01$, *** $p < 0.001$ and N.S., not significant ($p > 0.05$) by the unpaired *t*-test (**a-f**). Data are from representative of three independent experiments.

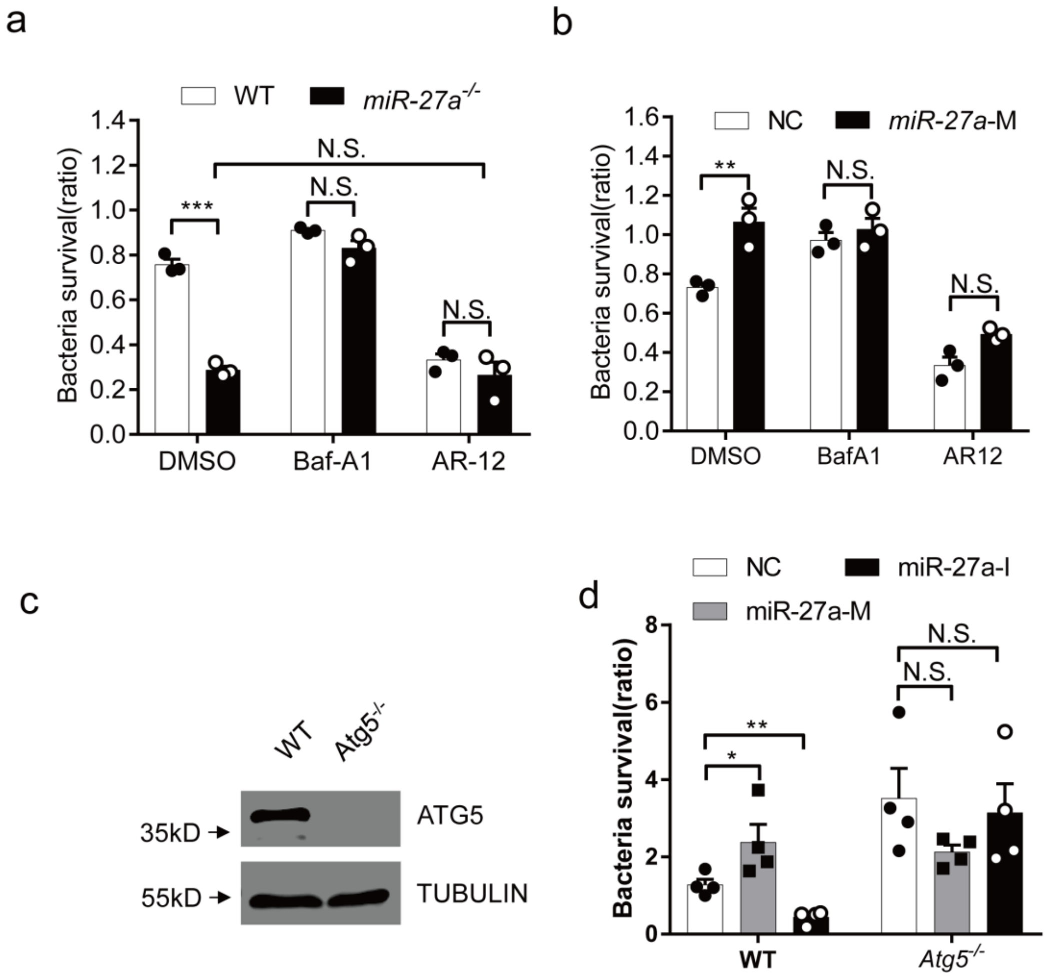

Supplementary Figure 3 Regulation of autophagy by miR-27a **(a)** Bacterial survival of *Mtb* in peritoneal macrophages from WT or *miR-27a*$^{-/-}$ mice treated with AR-12(10μM) or Bafilomycin A1 (100 nM) for 2 hours and then infected with *Mtb* (MOI=5) at 24 hours compared to 2 hours. (**b**) Bacterial survival of *Mtb* in peritoneal macrophages transfected with Negative control or miR-27a mimics, treated with AR-12(10μM) or Bafilomycin A1 (100 nM) for 2 hours and then infected with *Mtb* (MOI=5) at 24 hours compared to 2 hours.(**c**) Immunoblot of ATG5 of WT and *Atg5*$^{-/-}$ Raw264.7 macrophages. (**d**) Bacterial survival of *Mtb* in WT and *Atg5*$^{-/-}$ Raw264.7 macrophages infected with *Mtb* (MOI=5) at 24 hours compared to 2 hours. * $p < 0.05$, ** $p < 0.01$, *** $p < 0.001$ and N.S., not significant ($p > 0.05$) by the unpaired *t*-test (**a, b, d**). Data are from representative of three independent experiments.

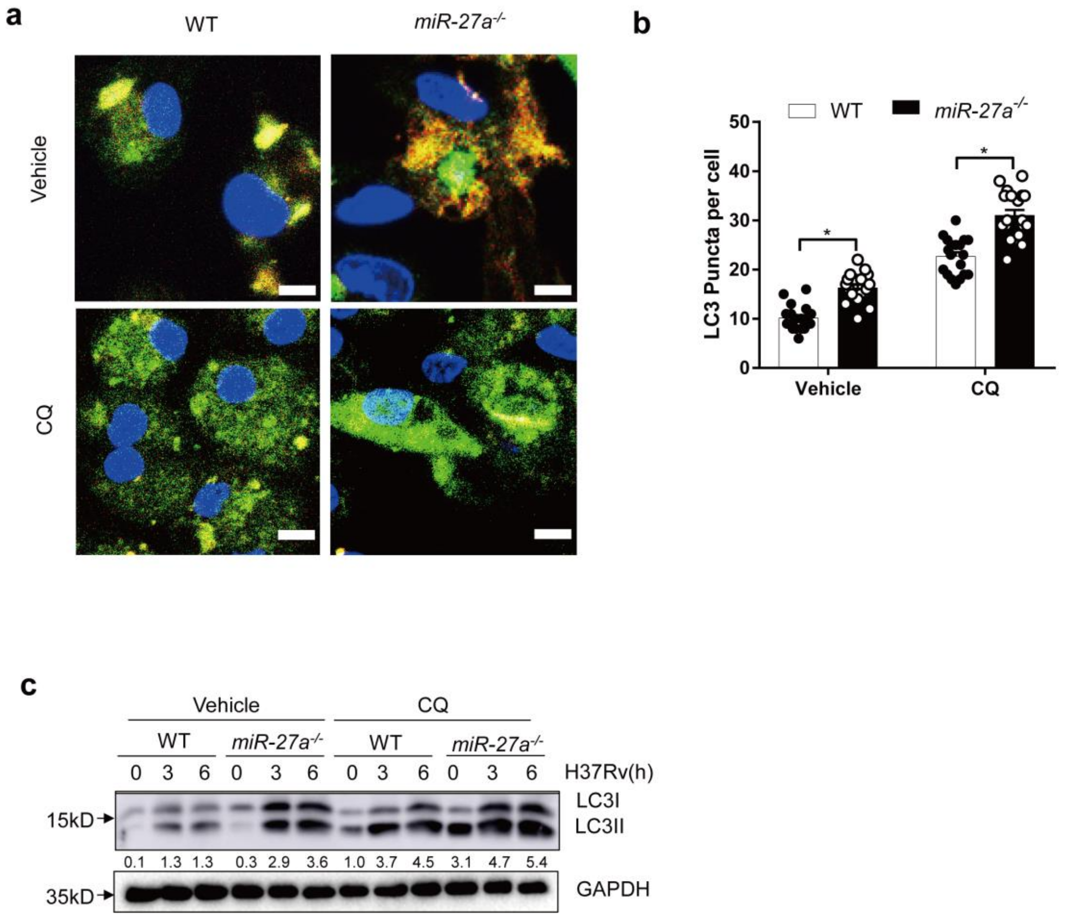

Supplementary Figure 4 miR-27a regulates autophagy. (a) Confocal analysis of mRFP-GFP-LC3B spots in WT or *miR-27a*$^{-/-}$ peritoneal macrophages treated with CQ(10ug/ml) for 4 hours and then infected with *Mtb* (MOI=5) for 6 hours. Bar, 7.5μM (**b**) Quantification of LC3 spots in WT or *miR-27a*$^{-/-}$ peritoneal macrophages treated with CQ(10μM) for 4 hours and then infected with *Mtb* (MOI=5) for 6 hours. (**c**) Immunoblot of LC3I/II in WT or *miR-27a*$^{-/-}$ macrophages pretreated with Vehicle or CQ(10μM) for 4 hours and then stimulated with *Mtb* at MOI 5 for indicated times. * $p < 0.05$ by the unpaired *t*-test (**b**). Data are from representative of three independent experiments.

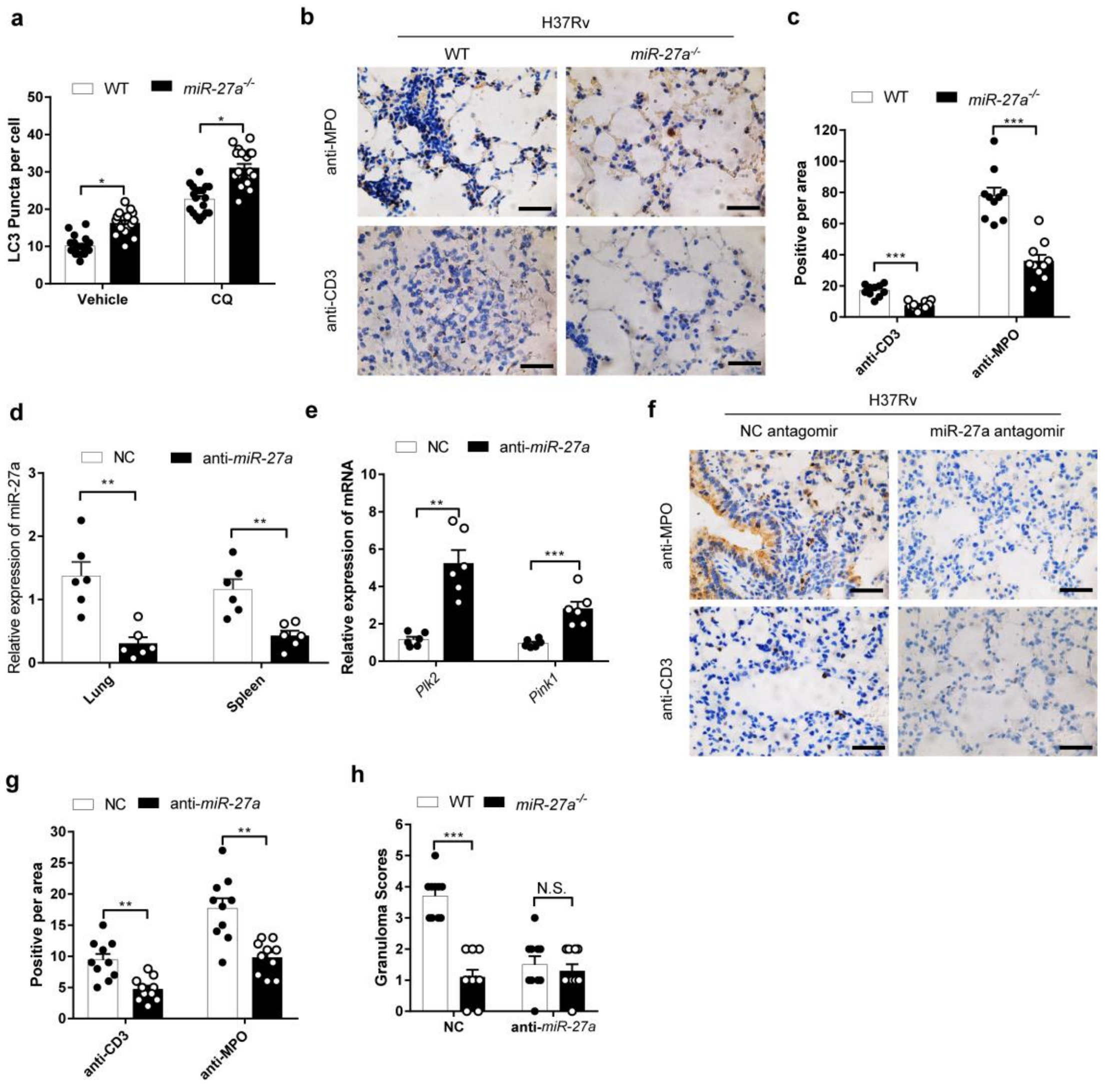

Supplementary Figure 5 miR-27a controls susceptibility to *Mtb*. (a) Quantification of granuloma scores in lung tissue section from WT or *miR-27a*$^{-/-}$ mice infected with *Mtb* H37Rv for 28 days. (**b**)Immuno-histochemical staining of anti-MPO or anti-CD3 in lung tissue section from WT or *miR-27a*$^{-/-}$ mice infected with *Mtb* H37Rv for 28 days. (**c**) Quantification of positive signals **of** anti-MPO or anti-CD3 in lung tissue section from WT or *miR-27a*$^{-/-}$ mice infected with *Mtb* H37Rv for 28 days. (**d**) Real-time PCR detection of miR-27a in lung tissues or spleen tissues of mice treated with miR-27a antagomir. (**e**) Real-time PCR detection of *Plk2* or *Pink1* in lung tissues of mice treated with miR-27a antagomir. (**f**) Immuno-histochemical staining of anti-MPO or anti-CD3 in lung tissue section from mice infected with *Mtb* H37Rv for 28 days and treated with miR-27a antagomir for 15 days. (**g**) Quantification of

positive signals of anti-MPO or anti-CD3 in lung tissue section from mice infected with *Mtb* H37Rv for 28 days and treated with miR-27a antagomir for 15 days. (**h**) Quantification of granuloma scores in lung tissue section from WT or *miR-27a*$^{-/-}$ mice infected with *Mtb* H37Rv for 28 days and treated with miR-27a antagomir for 15 days. ** p < 0.01 by the unpaired *t*-test (**a, c-e, g, h**). Data are from representative of three independent experiments.

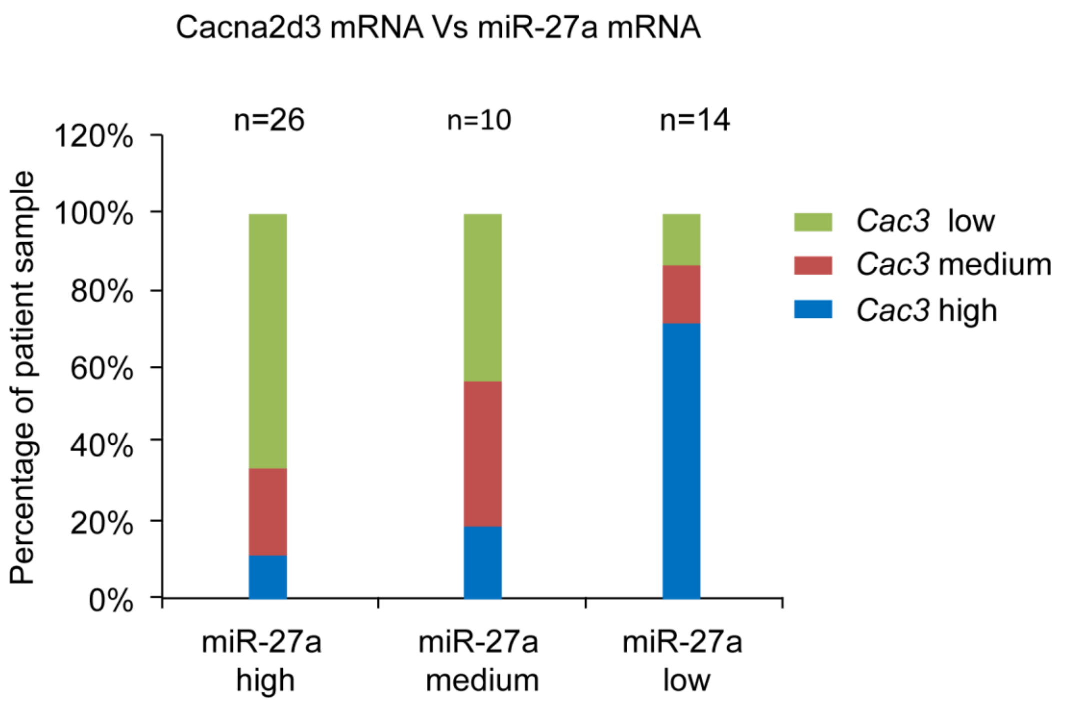

Supplementary Figure 6 Negative Correlation between the *Cacna2d3* and *miR-27a*. mRNA in PBMC from TB patients, both *miR-27a* and *Cacna2d3* levels were classified as low, medium, or high based on the real-time PCR, and the percentages of patients classified in each category are depicted in the histogram.

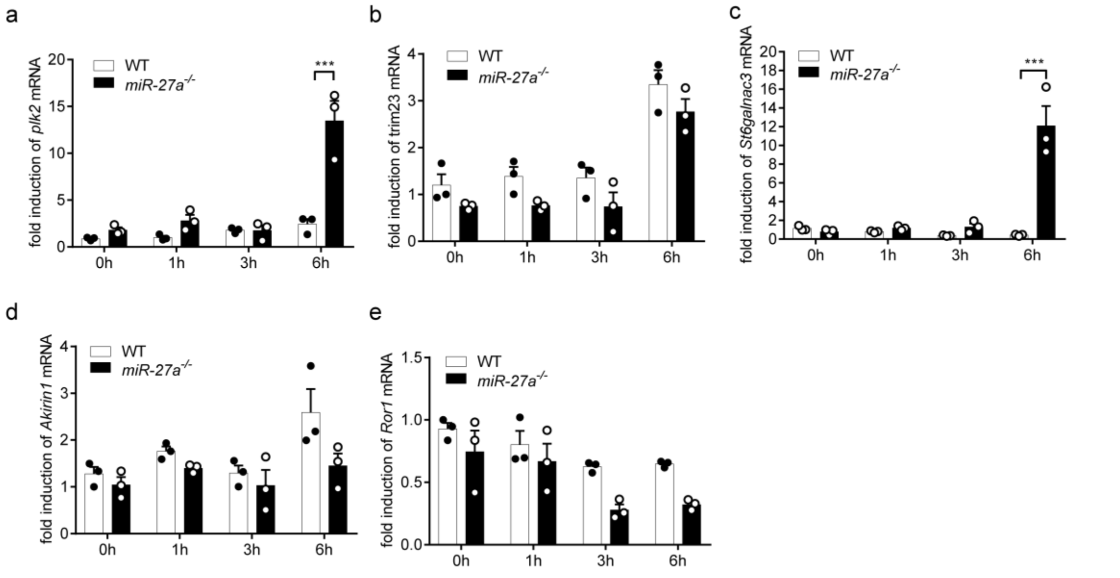

Supplementary Figure 7 miR-27a have multiple targets. (**a**)-(**e**)Real-time PCR detection of *Plk2* (**a**), *Trim23*(**b**), *St6galnac3* (**c**), *Akirin* (**d**) or *Ror1* **(e)** in WT or *miR-27a*$^{-/-}$ primary peritoneal macrophages infected with *Mtb* (MOI=5) at indicated times . *** p < 0.001 by the unpaired t-test (**a-e**). Data are from representative of three independent experiments.

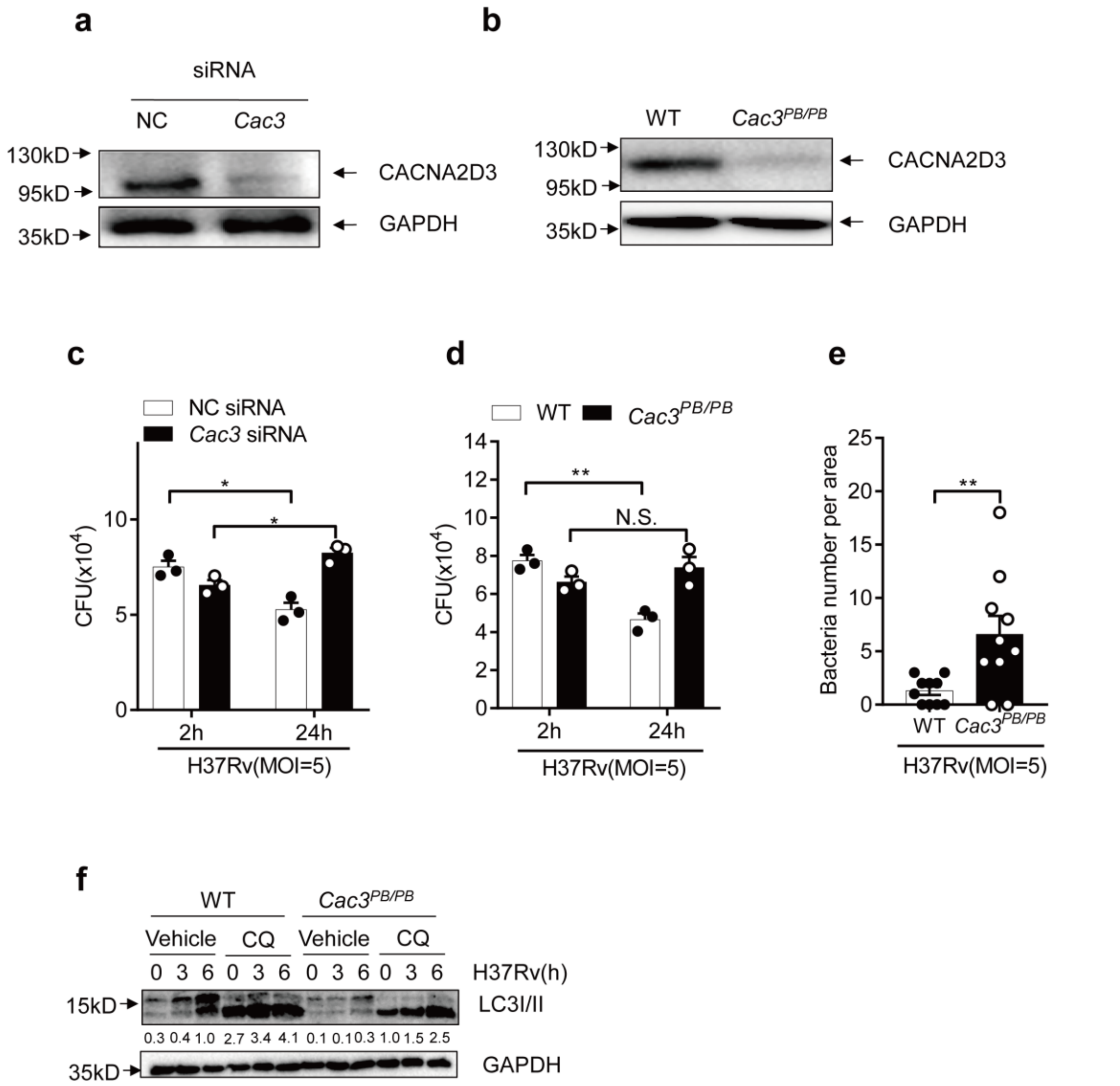

Supplementary Figure 8 *Cac3* regulate intracellular survival of *Mtb*. (a)&(b) Immunoblot of CACNA2D3 in macrophages pre-transfected with NC siRNA or *Cac3* siRNA for 48 hours. (**b**) Immunoblot of CACNA2D3 in WT or $Cac3^{PB/PB}$ macrophages. (**c**)CFU assay in primary peritoneal macrophages pretreated with NC siRNA or *Cac3* siRNA for 48 hours and then infected with *Mtb* (MOI=5) for 2 hours or 24 hours. (**d**) CFU assay in primary peritoneal macrophages from WT or $Cac3^{PB/PB}$ mice infected with *Mtb* (MOI=5) for 2 hours or 24 hours. (**e**) Bacterial numbers counted in each visual area in WT or $Cac3^{PB/PB}$ macrophages infected with *Mtb* (MOI=5) for 24 hours and then sent to TEM examination. (**f**) Immunoblot of LC3I/II in WT or $Cac3^{PB/PB}$ macrophages pretreated with Vehicle or CQ(10μM) for 4 hours and then stimulated with *Mtb* at MOI 5 for indicated times.* $p < 0.05$, ** $p < 0.01$,

and N.S., not significant (p >0.05) by the unpaired t-test (**c-e**). Data are from representative of three independent experiments.

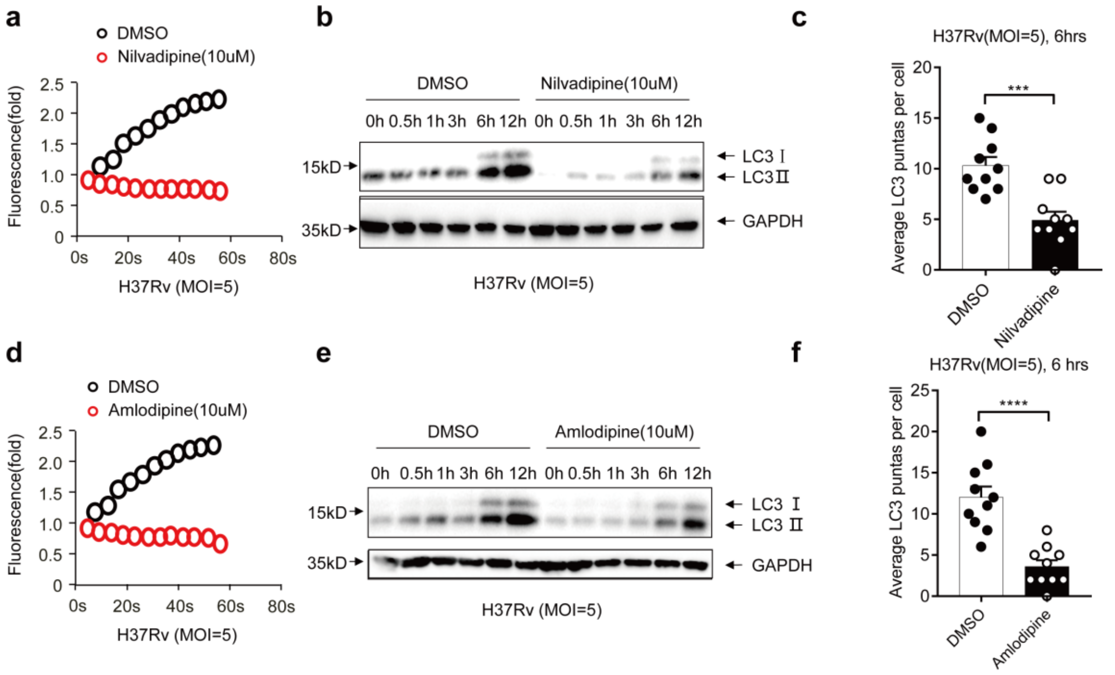

Supplementary Figure 9 Cacna2d3 regulates Ca^{2+} mediated-autophagy after *Mtb* infection (**a**) Intracellular calcium concentration detection in macrophages pretreated with DMSO or Nilvadipine(10μM) stimulated with *Mtb* at MOI 5 for the duration of 1 minute. (**b**) Immunoblot of LC3I/II in macrophages pretreated with DMSO or Nilvadipine(10μM) stimulated with *Mtb* at MOI 5 for indicated times. (c) A high-content imaging assay on the Cellomics ArrayScan platform to screen for LC3 spots in macrophages pretreated with DMSO or Nilvadipine(10μM) stimulated with *Mtb* at MOI 5 for 24 hours. (**d**)The intracellular Calcium concentration detection in macrophages pretreated with DMSO or Amlodipine(10μM) stimulated with *Mtb* at MOI 5 for the duration of 1 minute. (**e**) Immunoblot of LC3I/II in macrophages pretreated with DMSO or Amlodipine(10μM) stimulated with *Mtb* at MOI 5 for indicated times. (**f**)A high- content imaging assay on the Cellomics ArrayScan platform to screen for LC3 spots in macrophages pretreated with DMSO or Amlodipine(10μM) stimulated with *Mtb* at MOI 5 for 24 hours. ** $p < 0.01$ by the unpaired t-test (**c, f**). Data are representative of three independent experiments (**a, b, d,**

e).

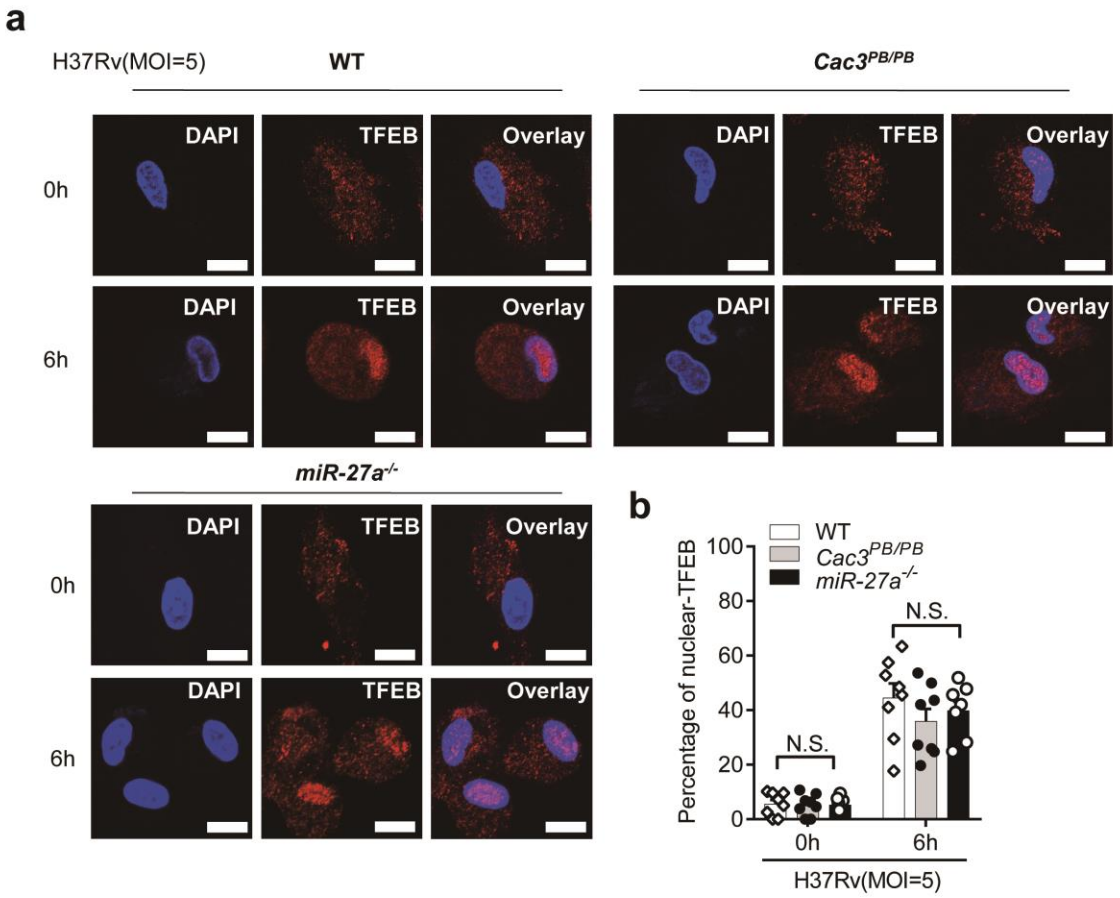

Supplementary Figure 10 Cacna2d3 regulates the autophagy partly through TFEB (**a**) confocal microscopy analysis of TFEB in peritoneal primary macrophages from WT, *Cac3*$^{PB/PB}$ or *miR-27a*$^{-/-}$ mice treated with *Mtb* H37Rv at MOI=5 for 6 hours. Scale Bar, 5 μM (**b**) Calculation of the average percentage of nuclear localization of TFEB. Data are representative of three independent experiments (**a**). N.S. stands for no significant difference by the unpaired *t*-test (**b**). Data are from representative of three independent experiments.

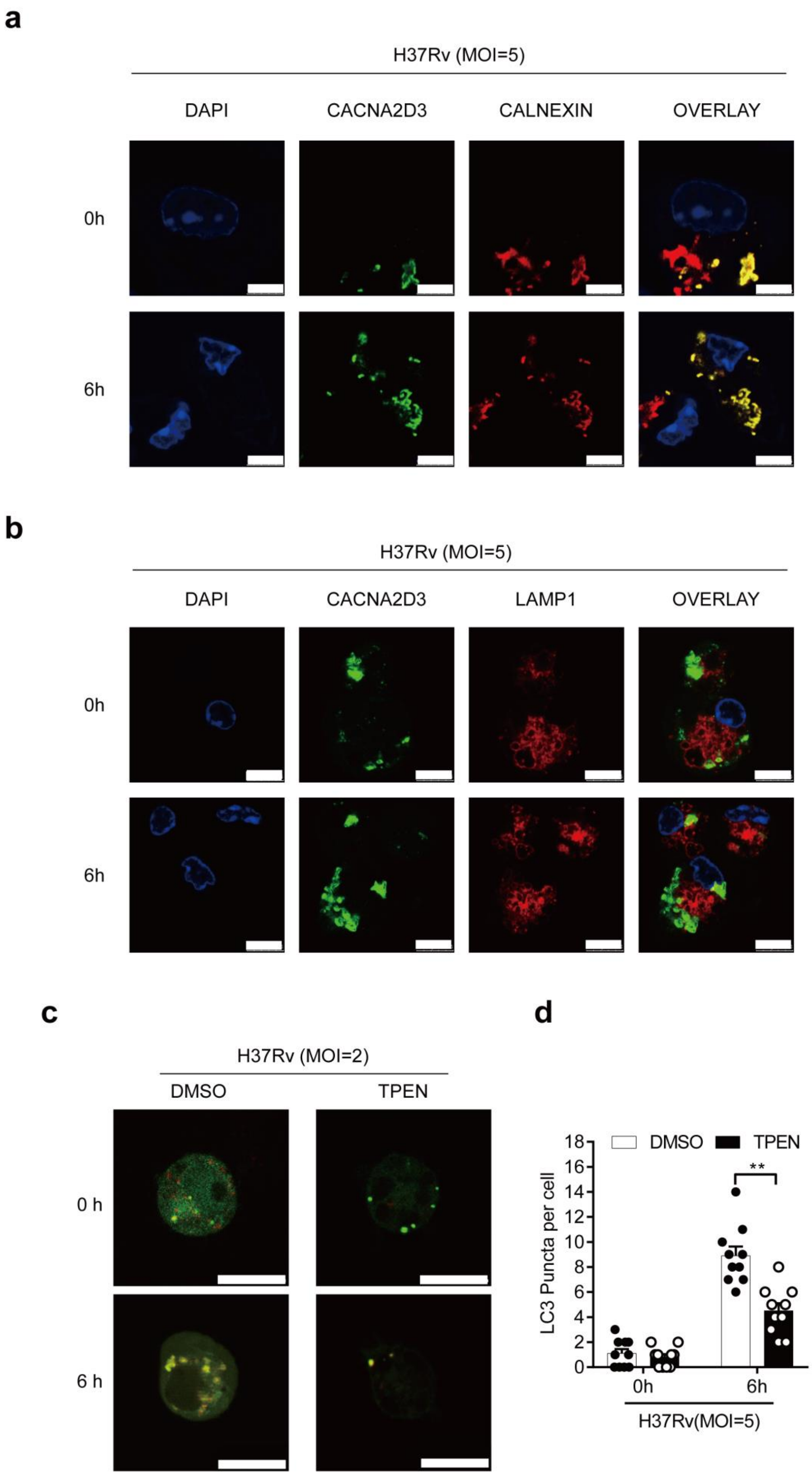
a
H37Rv (MOI=5)
DAPI
CACNA2D3
CALNEXIN
OVERLAY
0h
6h
b
H37Rv (MOI=5)
DAPI
CACNA2D3
LAMP1
OVERLAY
0h
6h
c
H37Rv (MOI=2)
DMSO
TPEN
0 h
6 h
d
DMSO
TPEN
**
LC3 Puncta per cell
0
2
4
6
8
10
12
14
16
18
0h
6h
H37Rv(MOI=5)

Supplementary Figure 11 Cacna2d3 co-localizates on ER membrane and regulates Ca^{2+}-induced autophagy in H37Rv infection (**a**) Confocal microscopy analysis of CALNEXIN and CACNA2D3 in macrophages infected with *Mtb* at MOI=5 for 6 hours. Scale Bar, 5 μM (**b**) Confocal microscopy analysis of LAMP1 and CNACA2D3 in macrophages infected with *Mtb* at MOI=5 for 6 hours. Scale Bar, 7.5 μM (**c**) Confocal microscopy analysis of mRFP-GFP-LC3 in macrophages pretreated with vehicle or ER calcium chelator PTEN (50uM) for 30 min and then infected with *Mtb* H37Rv for 6 hours. Scale Bar, 10 μM (**d**) Calculation of the average of LC3 spots in each cell. ** p < 0.01 by the unpaired t-test(**d**). Data are representative of three independent experiments (**a, b, c**).

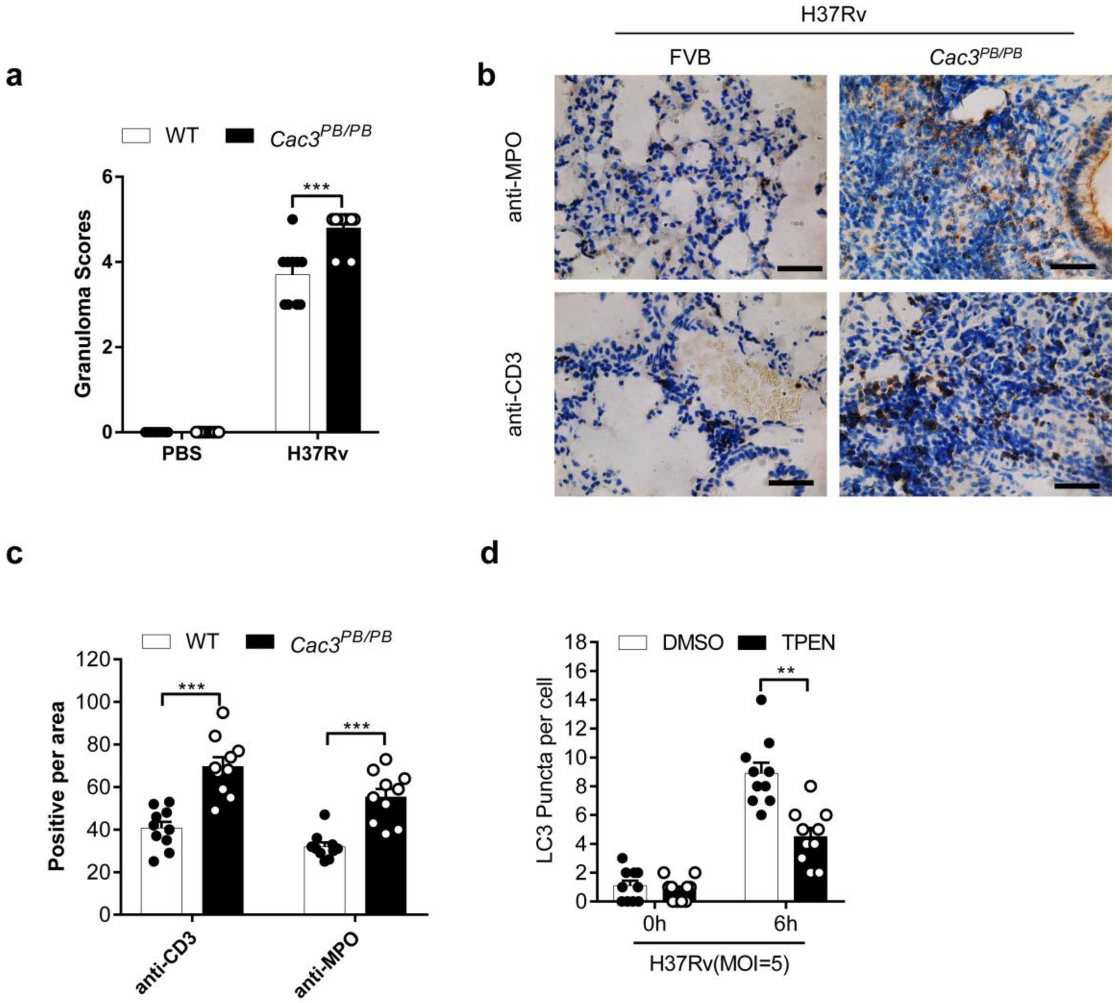

Supplementary Figure 12 Cacna2d3 controls susceptibility to *Mtb*. (a)

Quantification of granuloma scores of H&E staining of lung tissue section from WT or $\mathrm{Cac3}^{PB/PB}$ mice infected with *Mtb* H37Rv for 28 days. **(b)**Immuno-histochemical staining of anti-MPO or anti-CD3 in lung tissue section from WT or $Cac3^{PB/PB}$ mice infected with *Mtb* H37Rv for 28 days. **(c)** Quantification of positive signals of anti-MPO or anti-CD3 in lung tissue section from WT or $Cac3^{PB/PB}$ mice infected with *Mtb* H37Rv for 28 days. (**d**) Quantification of granuloma scores in lung tissue section from WT or $Cac3^{PB/PB}$ mice infected with *Mtb* H37Rv for 28 days and treated with miR-27a antagomir for 15 days. *** $p < 0.001$ by the unpaired *t*-test (**a, c, d**). Data are from representative of three independent experiments.

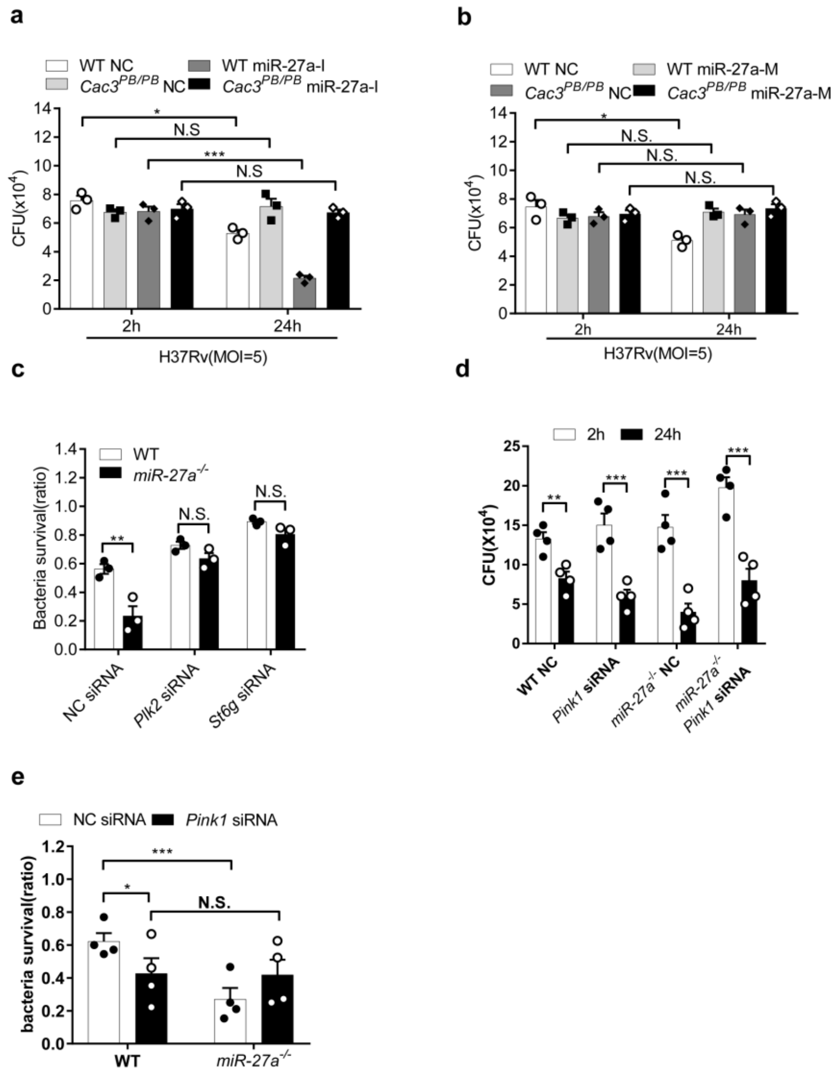

Supplementary Figure 13 miR-27a regulates intracellular survival of *Mtb* via *Cacna2d3*. CFU assay in primary peritoneal macrophages from WT or *Cac3* $^{PB/PB}$ mice pre-transfeted with miR-27a mimic(**a**) or miR-27a inhibitor(**b**) for 48 hours and then infected with *Mtb* (MOI=5) for 2 hours or 24 hours. (**c**) Bacterial survival of *Mtb* in macrophages transfected with *Plk2* siRNA or *St6galnac3* siRNA for 48 hours and then infected with *Mtb* (MOI=5) at 24 hours. (**d**) CFU assay in primary peritoneal macrophages from WT or *miR-27a*$^{-/-}$ mice transfected with *Pink1* siRNA for 48 hours and then infected with *Mtb* (MOI=5) for 2 hours or 24 hours. (**e**) Bacterial survival of *Mtb* in macrophages transfected with *Pink1* siRNA for 48 hours and then infected with *Mtb* (MOI=5) at 24 hours. * $p < 0.05$, ** $p < 0.01$, *** $p < 0.001$, and N.S., not significant ($p > 0.05$) by the unpaired *t*-test (**a-e**)

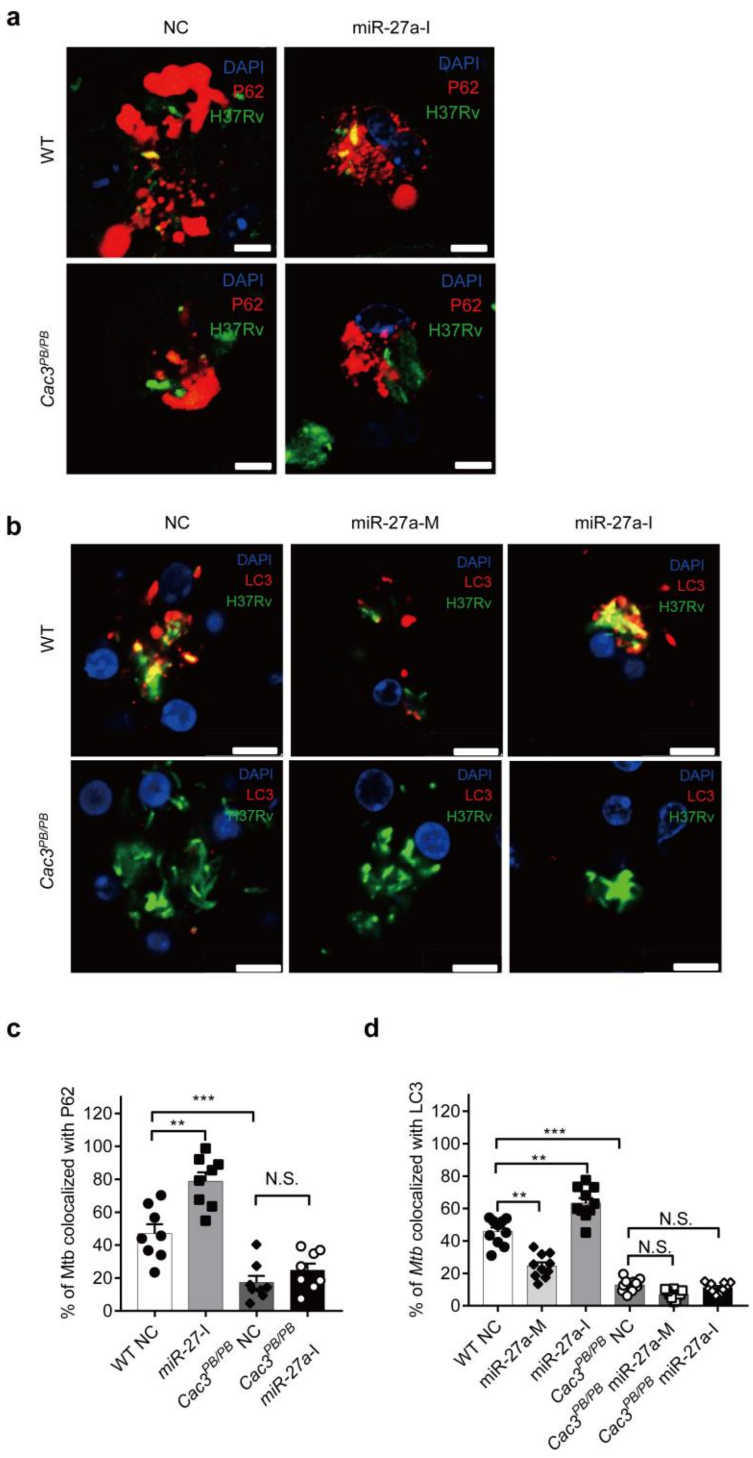

Supplementary Figure 14 miR-27a regulates xenophagy of *Mtb* via *Cacna2d3*. (**a**) Adenovirus-mcheery-P62 harboring peritoneal macrophages from WT and *Cac3* $^{PB/PB}$ mice were transfected with miR-27a inhibitors for 48 hours, infected with FITC-labelled *Mtb* H37Rv, and then sent to confocal analysis. Scale Bar, 7.5 μM (**b**)Immunostaining of LC3 and confocal analysis of peritoneal macrophages from WT and *Cac3* $^{PB/PB}$ mice were transfected with miR-27a inhibitors or mimics for 48 hours,

and then infected with FITC-labelled *Mtb* H37Rv. Scale Bar, 5 μM (**c**) Quantification of percentage of P62-positive *Mtb*. (**d**) Quantification of percentage of LC3-positive *Mtb*. ** $p < 0.05$, *** $p < 0.001$, and N.S., not significant ($p > 0.05$) by the unpaired *t*-test (**c,d**). Data are representative of three independent experiments (**a, b, c, d**).

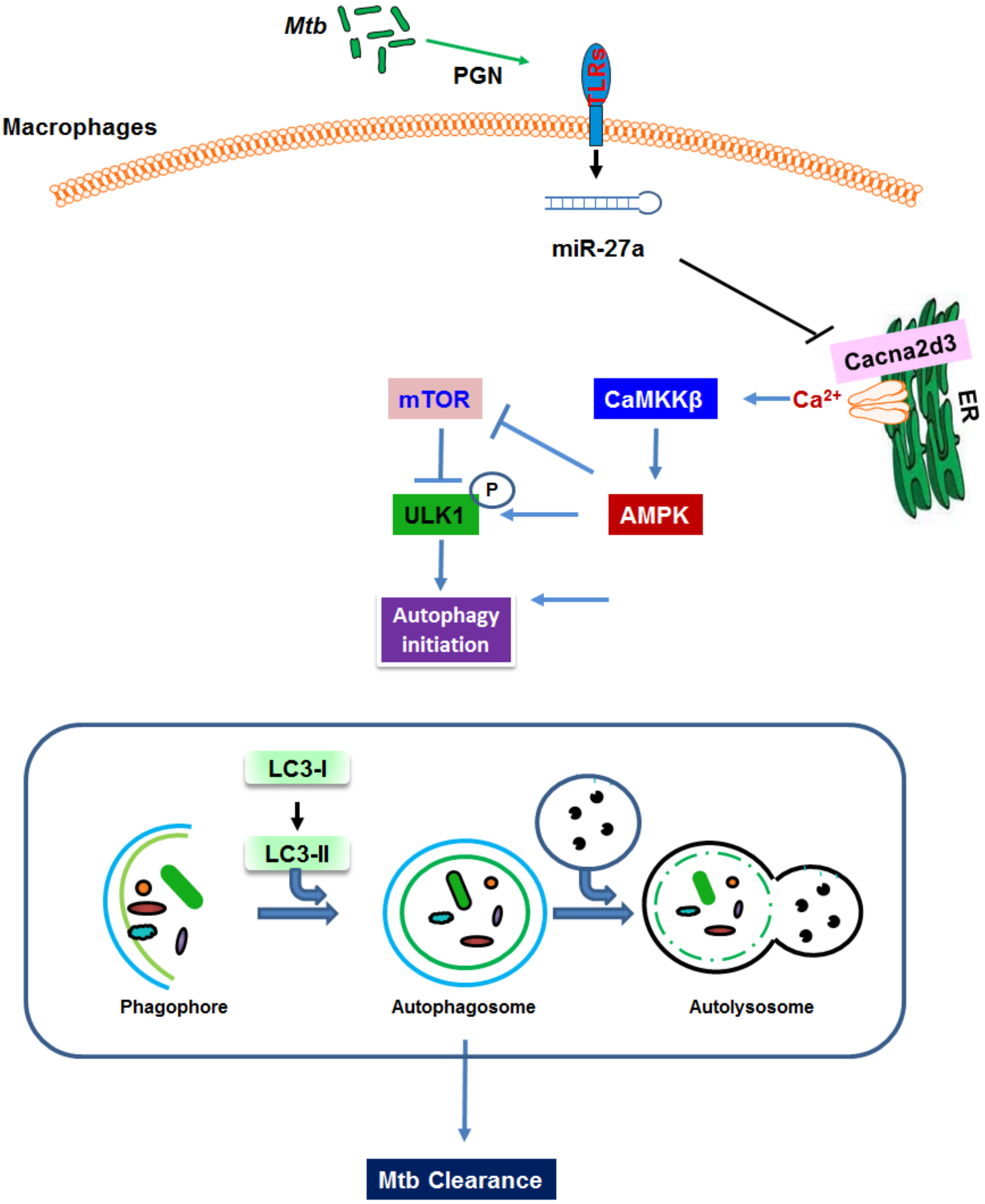

Supplementary Figure 15 A diagram depicting the functional role of miR-27a via Cacna2d3 in the regulation of Ca^{2+}-induced autophagy in H37Rv infection.

Supplementary Figure 16

Original WB figures

Figure 1f

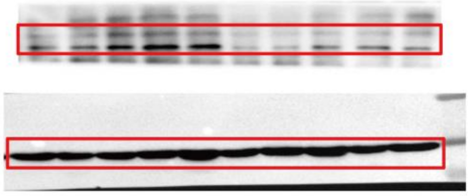

LC3I/II

GAPDH

Figure 1g

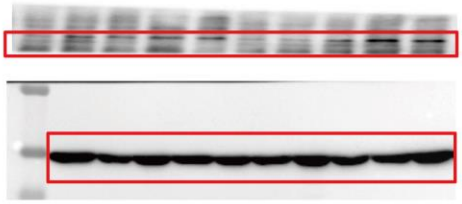

LC3I/II

GAPDH

Figure 1j

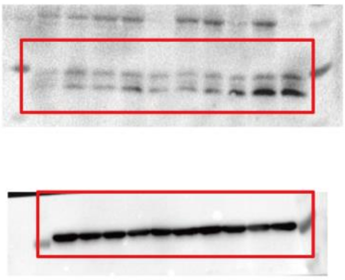

LC3I/II

GAPDH

Figure 3i

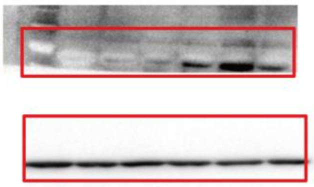

CACNA2D
3

GAPDH

Figure 3g, h

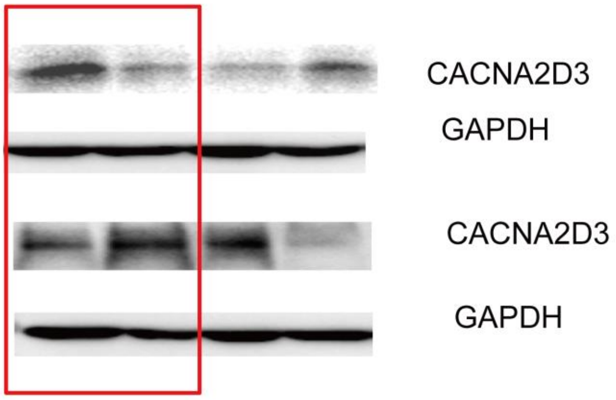

Figure 4d

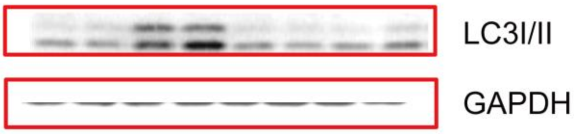

Figure 4n

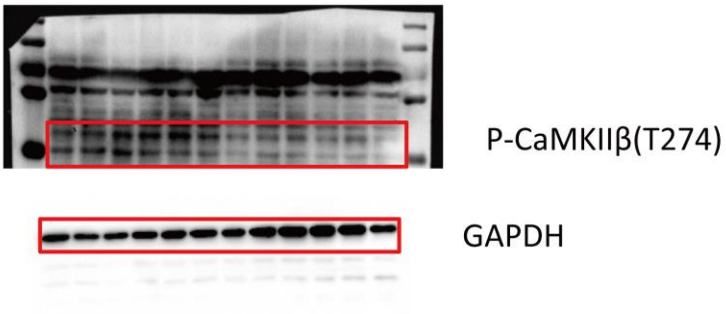

Figure 4o

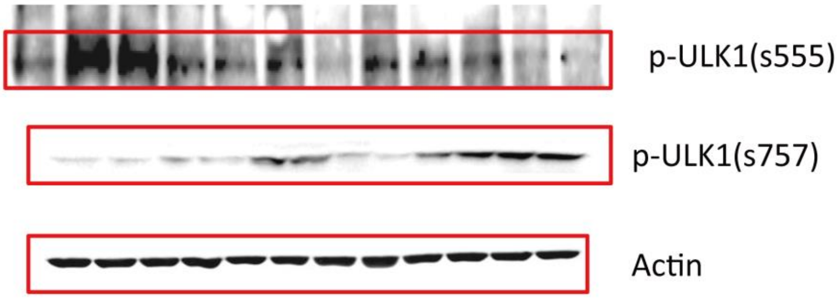

Supplementary Fig. 3c

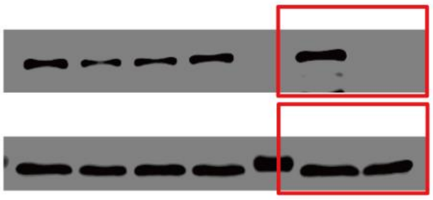

Supplementary Fig. 4c

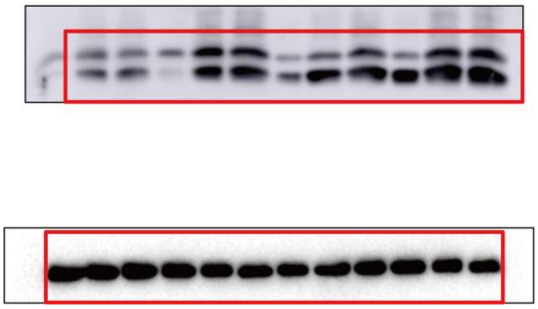

Supplementary Figure 8a

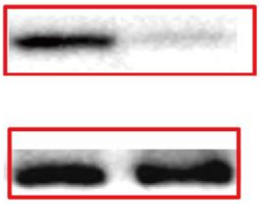

Supplementary Figure 8b

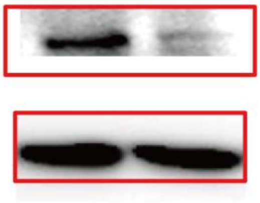

Supplementary Figure 8f

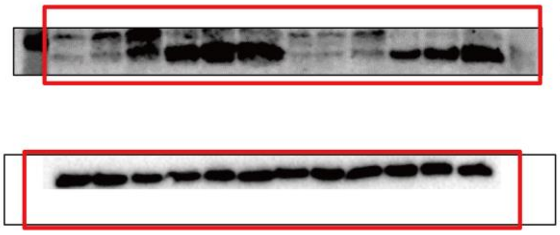

Supplementary Figure 9b

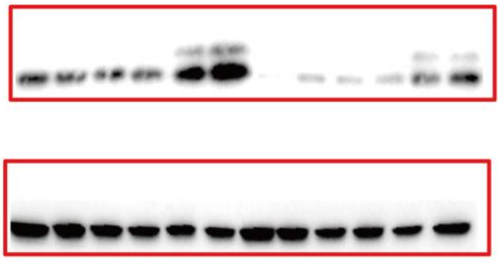

Supplementary Figure 9e

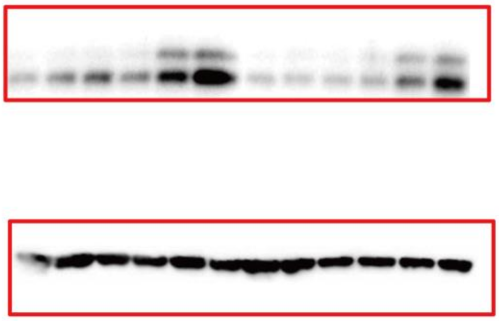

Supplementary Table 1. Realtime PCR Primers

human CACNA2D3	
forward	GCCTGTGAACATCAGTCTAAGTG
reverse	ATCCCCGGATACTGCCTAAAA
human GAPDH	
forward	GGAGCGAGATCCCTCCAAAAT
reverse	GGCTGTTGTCATACTTCTCATGG
mouse Cacna2d3	
forward	CCAGGGATTAAATGGGAACCAG
reverse	ACGTCCACCAAAATGACAACA
mouse Tfeb	
forward	AAGGTTCGGGAGTATCTGTCTG
reverse	GGGTTGGAGCTGATATGTAGCA
mouse Pink1	
forward	TTCTTCCGCCAGTCGGTAG
reverse	CTGCTTCTCCTCGATCAGCC
mouse Plk2	
forward	CCTGCGGACTATCACCTACCA
reverse	CTGCCCATCTTCAGAAGGCT
mouse Ror1	
forward	TGAGCCGATGAATAACATCACAA
reverse	CAGGTGCATCATTCTTGAACCA
mouse Akirin1	
forward	GAGCGTCGTCTTCCAACTCC
reverse	GAAGTACAGGCTTCGCTTTGA
mouse Stg6nac3	
forward	TGTGGTGAGCTTCATAGCCCT
reverse	GATCCATTTGGTCTTAGGCTGTC
mouse Trim23	
forward	ACCAGAAGCTAATCAGATCCGA
reverse	TCTGTGTGAGCCATTCCAATTC
mouse Gapdh	
forward	GGAGCGAGATCCCTCCAAAAT
reverse	GGCTGTTGTCATACTTCTCATGG
miRNA q-PCR kit	Tiangen Accession No.
miR-23a	CD201-0311
miR-24	CD202-0009
miR-27a	CD202-0033

Supplementary Table 2 siRNA sequence

mouse Plk2 pool	
oligo1	CCGAGATCTCGCGGATTAT
oligo2	GCAAGGGTGGATTTGCAAA
oligo3	GGTCCATGGCTCACATCTT
mouse St6galnac3 pool	
oligo1	GCTTGTGGTGAGCTTCATA
oligo2	GCATCTTGCTGCTAGCCAT
oligo3	GCCTCTTCGAACTCACTAT
mouse Pink1 pool	
oligo1	GGATCCAGAGGCAGTTCAT
oligo2	CCATTGGCAAGGGTTGCAA
oligo3	CCAAACACCTTGGCCTTAT
